# Cerebrovascular Injury After Serial Exposure to Chronic Stress and Abstinence from Methamphetamine Self-Administration

**DOI:** 10.1038/s41598-018-28970-1

**Published:** 2018-07-12

**Authors:** Reka Natarajan, Carmen M. Mitchell, Nicole Harless, Bryan K. Yamamoto

**Affiliations:** 1 Department of Pharmacology and Toxicology, Indiana University School of Medicine 635 Barnhill Drive MS A401, Indianapolis, IN 46202 USA; 2 Department of Neurosciences, University of Toledo College of Medicine 3000 Arlington Avenue MS 1007, Toledo, OH 43614 Spain

## Abstract

Cerebrovascular damage caused by either exposure to stress or the widely abused drug, methamphetamine (Meth) is known but stress and drug abuse frequently occur in tandem that may impact their individual cerebrovascular effects. This study examined their co-morbid cerebrovascular effects during abstinence from self-administered Meth after the exposure to chronic unpredictable stress (CUS). Exposure to CUS prior to unrestricted Meth self-administration had no effect on Meth intake in rats; however, the pro-inflammatory mediator cyclooxygenase-2 (COX-2) and the breakdown of cell-matrix adhesion protein β-dystroglycan in isolated cerebral cortical capillaries were increased after 3 days of abstinence and persisted for 7 days. These changes preceded decreases in occludin, a key structural protein component of the blood-brain barrier. The decrease in occludin was blocked by the COX-2 specific inhibitor nimesulide treatment during abstinence from Meth. The changes in COX-2, β-dystroglycan, and occludin were only evident following the serial exposure to stress and Meth but not after either one alone. These results suggest that stress and voluntary Meth intake can synergize and disrupt cerebrovasculature in a time-dependent manner during abstinence from chronic stress and Meth. Furthermore, COX-2 inhibition may be a viable pharmacological intervention to block vascular changes after Meth exposure.

## Introduction

Cerebrovascular injury has been reported in multiple neuropathologies including neurodegenerative and psychiatric diseases such as Parkinson’s disease, multiple sclerosis, depression, schizophrenia, and drug abuse^1–3^. The resultant effects of cerebrovascular injury include the entry of large endogenous immune molecules such as a immunoglobulins, macrophages and pro-inflammatory mediators derived from cyclooxygenase-2 (COX-2) into the brain that in turn, produce inflammation^4–6^ and cause oxidative damage, vascular injury and neurotoxicity^7^.

Inflammation in the periphery and the central nervous system are also caused by chronic exposure to stress^8–11^. In fact, stress exposure or treatment with the stress-associated hormone, corticosterone (CORT) activates microglia and causes vascular toxicity^12,13^. Furthermore, both acute and chronic exposure to stress increase microglial activation and cyclooxygenase-2 (COX-2) within the brain^14–16^ suggesting a role for inflammation in mediating cerebrovascular toxicity produced by stress.

Neuroinflammation is also observed after exposure to the widely abused drug, methamphetamine (Meth)^16^. Meth is a psychostimulant that causes mood and cognitive dysfunction attributed to monoaminergic neurodegeneration^17^. The neurodegenerative effects of Meth result in part from inflammation initiated by the activation of astrocytes and microglia, increased production of COX-2, and consequent increases in proinflammatory prostaglandins and cytokines such as TNF-α, IL1-β, and IL-6^18^. Moreover, Meth causes brain edema and extravasation of albumin into the brain parenchyma that are indicative of vascular permeability^5,19^. Regardless of whether cerebrovascular toxicity is produced by Meth alone or stress alone, stress and drug abuse occur frequently in tandem^20^ and may worsen the impact of their individual toxicities. In fact, stress or CORT prior to Meth injections causes greater long-term depletions of dopamine and serotonin, decreases in the structural proteins of the blood-brain barrier (BBB), occludin and β-dystroglycan, and increases in BBB permeability compared to Meth alone without prior exposure to stress^13,21–24^.

Prior studies have shown that Meth-induced damage to the BBB is blocked by ketoprofen, a non-specific COX inhibitor that blocks both constitutively expressed COX-1 and inducible COX-2^13,25^. Whereas COX-1 has been associated classically with homeostatic function and is not necessarily linked with inflammation, COX-2 is closely linked with inflammation processes and neurotoxicity^26,27^. Therefore, a direct assessment of COX-2 in mediating BBB changes due to inflammation per se is needed.

While protective strategies are typically introduced prophylactically prior to the onset of stress or Meth, it is unknown if specific anti-inflammatory interventions such as COX-2 antagonism subsequent to these insults would provide a feasible intervention strategy with a neuroprotective benefit. In addition, previous studies have used high doses of Meth that were administered non-contingently by the investigator over a 6 hour period prior to the assessment of cerebrovascular alterations. However, a more translational paradigm that approximates voluntary Meth intake behavior by humans and the “binge-crash” pattern typical of Meth use is needed^28^. Therefore, we modeled the serial exposure to varied, unpredictable stressors in conjunction with voluntary drug self-administration to examine if chronic unpredictable stress and self-administered Meth interact to alter the cerebrovasculature in a manner blocked by selective COX-2 antagonism during forced abstinence from Meth.

## Results

### Meth Self-Administration Behavior

**Figure 1:**
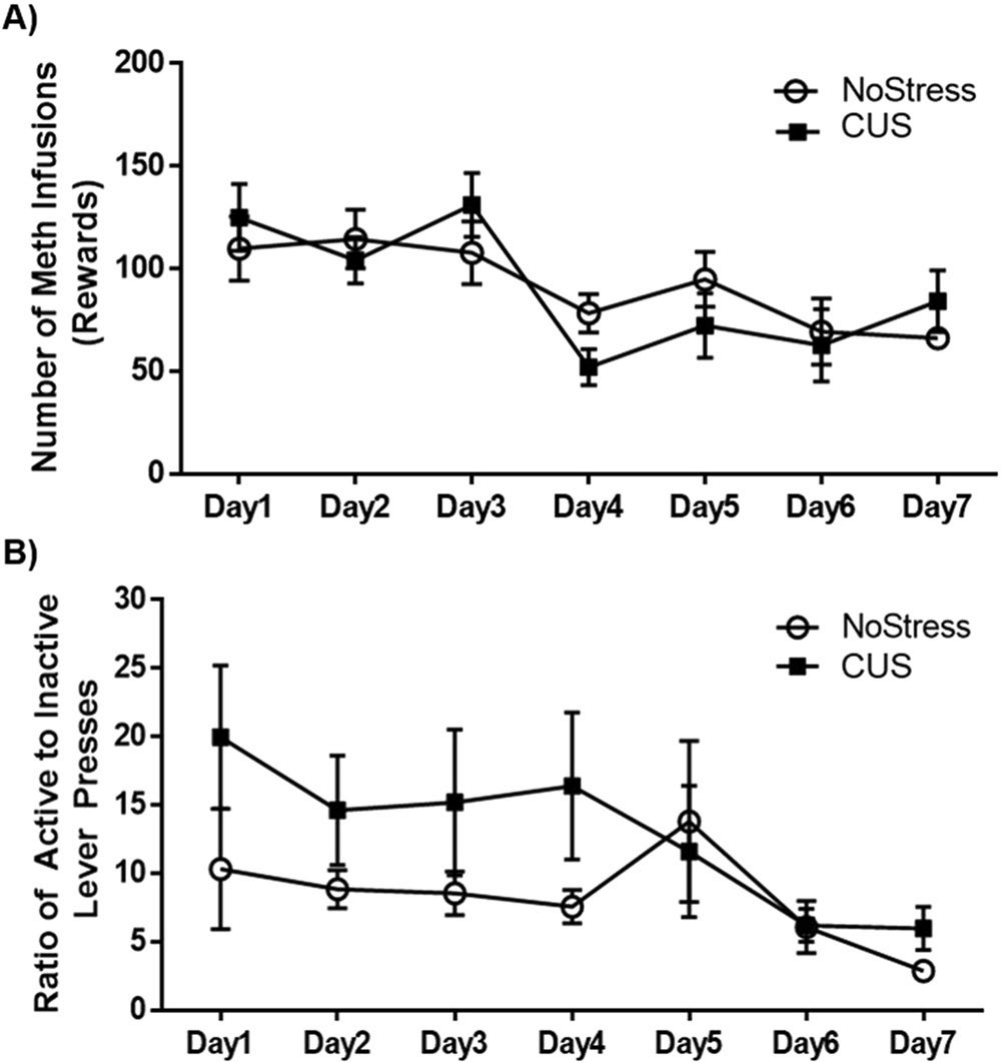
Meth self-administration behavior. (**A**) Rate of Meth intake between NoStress and CUS rats was not altered by prior stress exposure n = 27–29/group. (**B**) Ratio of active to inactive lever presses over the course of 7 days showed no difference between NoStress and CUS groups n = 27–29/group.

Meth self-administration behavior was assessed in rats exposed to 10 days of CUS. No differences were observed in the number of rewards received over the 7 days between the NoStress and CUS groups as there was no interaction between Stress and Day of self- administration (Fig. 1A). However, a main effect of Day was observed (F_(6,212)_ = 3.23, p < 0.01) with a decrease in lever presses over the course of 7 days. Additionally, no differences were observed in the ratio of active to inactive lever presses between CUS and NoStress groups or across the days of Meth self-administration (Fig. 1B).

### COX-2 in Cortical Capillaries

**Figure 2:**
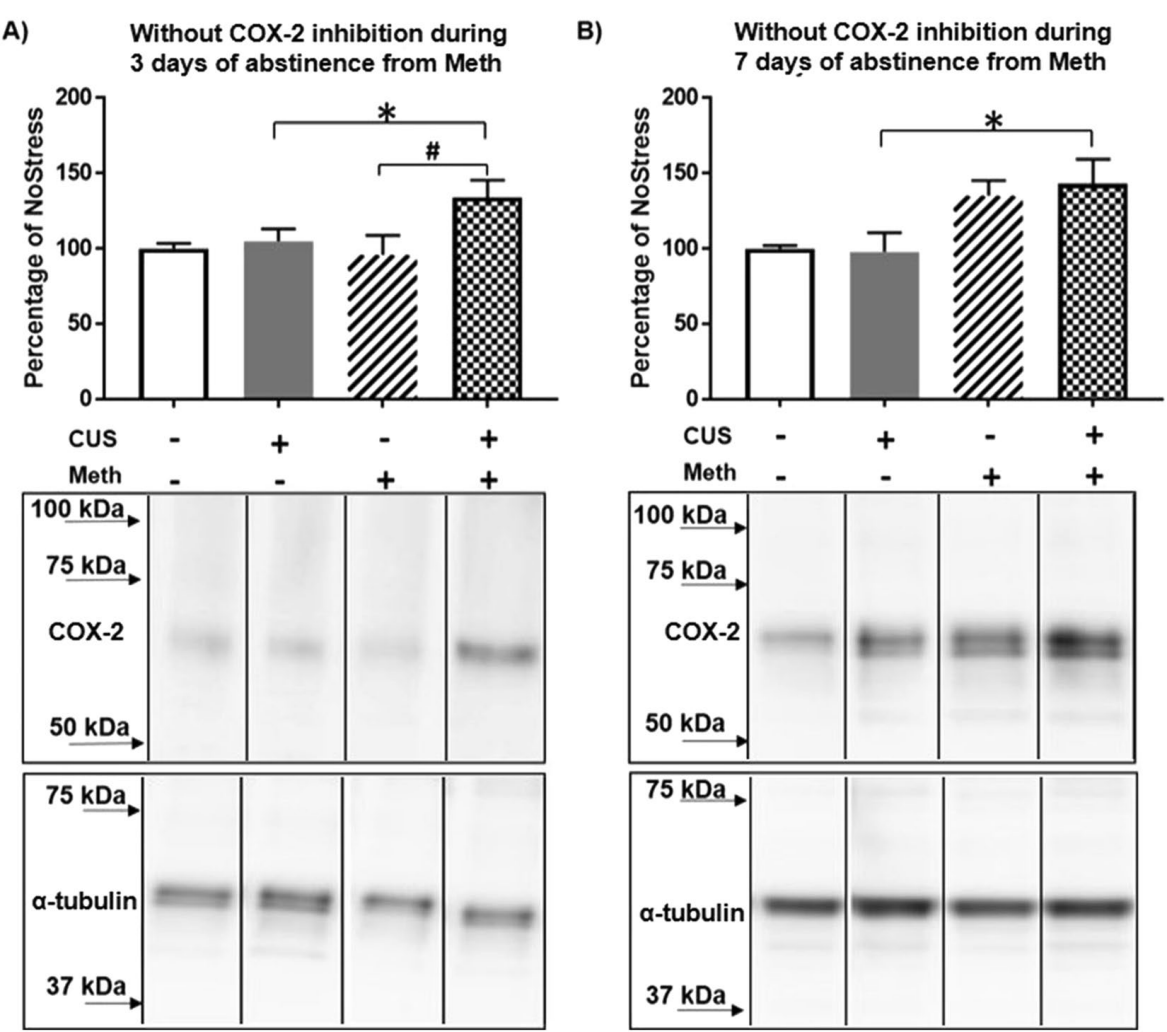
COX-2 in cortical microvasculature during abstinence from Meth. (**A)** 3 days of abstinence from Meth increased COX-2 in CUS + Meth compared to CUS. (*p < 0.05) and Meth (^#^p < 0.01) n = 6–8/group. Middle panel shows representative blot probed for COX-2 after 3 days of abstinence from Meth and the bottom panel shows the loading control α-tubulin. (**B)** 7 days of abstinence from Meth increased COX-2 in CUS + Meth compared to CUS (*p < 0.05) n = 6–8/group. Middle panel shows representative blot probed for COX-2 after 7 days of abstinence from Meth and the bottom panel shows the blot for loading control α-tubulin. COX-2 and α-tubulin band images presented have been vertically sliced in order to juxtapose lanes that were non-adjacent in the blot. Full-length blots are presented in Supplementary Figures S2A and S2B.

COX-2 protein assessed at 3 days after abstinence from Meth was generally higher in the CUS groups (F_(1,24)_ = 5.50, p < 0.05). Specifically, the increase in COX-2 in the CUS + Meth group was higher than the CUS group not exposed to Meth (q = 3.19, p < 0.05 CUS + NoMeth = 104.88 ± 9.11, CUS + Meth = 133.96 ± 9.11) and compared to the Meth groups without stress (NoStress + Meth (q = 4.02, p < 0.01 NoStress + Meth = 95.82 ± 9.84) (Fig. 2A). At 7 days after abstinence from Meth, COX-2 was increased overall (F_(1,24)_ = 10.43, p < 0.01) and in particular, CUS + Meth had higher levels of COX-2 compared to CUS + NoMeth (q = 3.63, p < 0.05 CUS + NoMeth = 98.14 ± 13.25, CUS + Meth = 143.13 ± 11.47) (Fig. 2B).

### Occludin in Cortical Capillaries

**Figure 3:**
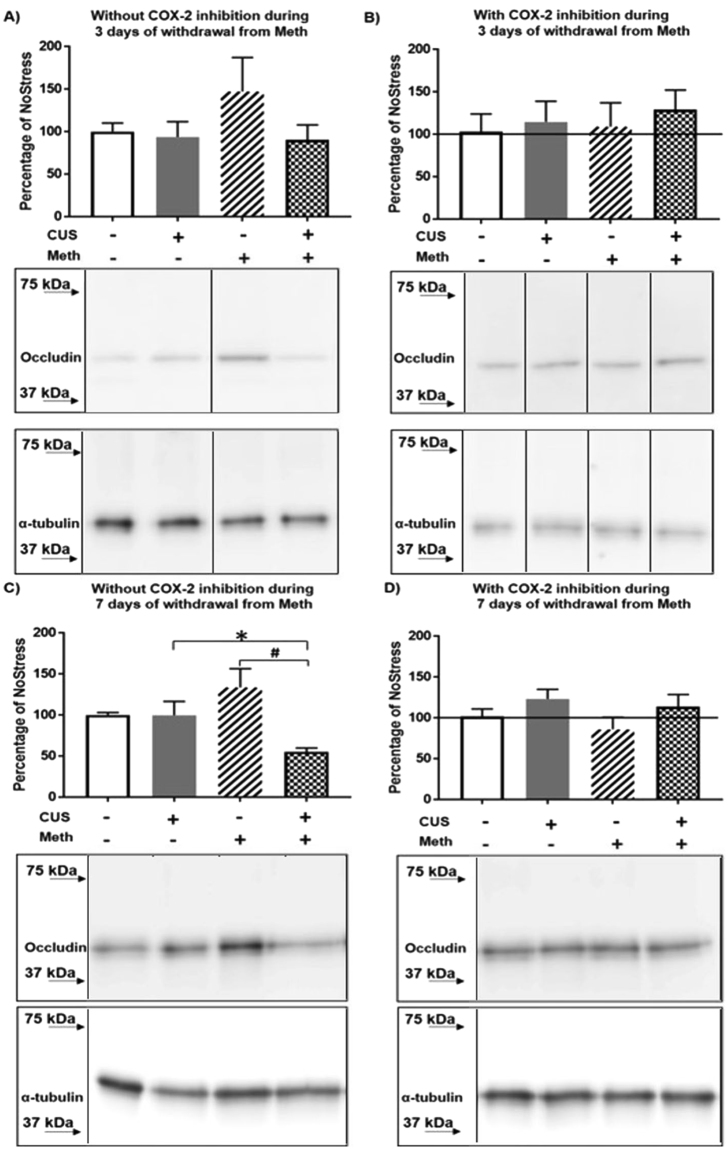
Occludin in cortical microvasculature during abstinence from Meth. (**A**) 3 days of abstinence from Meth. Middle panel shows representative blot of occludin after 3 days of abstinence from Meth and the bottom panel shows the loading control α-tubulin. (**B)** COX-2 inhibitor nimesulide treatment during 3 days of abstinence. Line indicates controls that did not receive nimesulide treatments. Middle panel shows a representative blot of occludin in nimesulide treated groups after 3 days of abstinence from Meth and the bottom panel shows the loading control α-tubulin. (**C**) 7 days of abstinence from Meth decreased occludin in CUS + Meth vs. CUS (*p < 0.05) and Meth (^#^p < 0.001) n = 6–8/group. Middle panel shows representative blot of occludin after 7 days of abstinence from Meth and the bottom panel shows the loading control α-tubulin. (**D)** COX-2 inhibitor nimesulide treatment during 7 days of abstinence. Line indicates controls that did not receive nimesulide treatments. Middle panel shows representative blot of occludin in nimesulide treated groups after 7 days of abstinence from Meth and the bottom panel shows the loading control α-tubulin. Occludin and α-tubulin band images presented have been vertically sliced in order to juxtapose lanes that were non-adjacent in the blot. Full-length blots are presented in Supplementary Figures S3A, S3B, S3C and D.

Changes in occludin were examined in the cortical vasculature 3 days after Meth abstinence with and without nimesulide treatment after Meth. No significant changes were observed in any group at 3 days (Fig. 3A,B). However, at 7 days of Meth abstinence, CUS produced an overall decrease in occludin (F_(1,24)_ = 7.93, p < 0.05) such that the decreases was greater in the Meth groups (F_(1,24)_ = 7.83, p < 0.05). Occludin was decreased more in the CUS + Meth group than in the CUS + NoMeth (q = 3.18, p < 0.05 CUS + NoMeth = 99.75 ± 14.99, CUS + Meth = 55.21 ± 12.98) and NoStress + Meth groups (q = 5.83, p < 0.001 NoStress + Meth = 133.52 ± 13.87) (Fig. 3C). Treatment with nimesulide during the 7 days of Meth abstinence blocked the decreases in occludin (Fig. 3D).

### Truncation of β-dystroglycan in Cortical Capillaries

**Figure 4:**
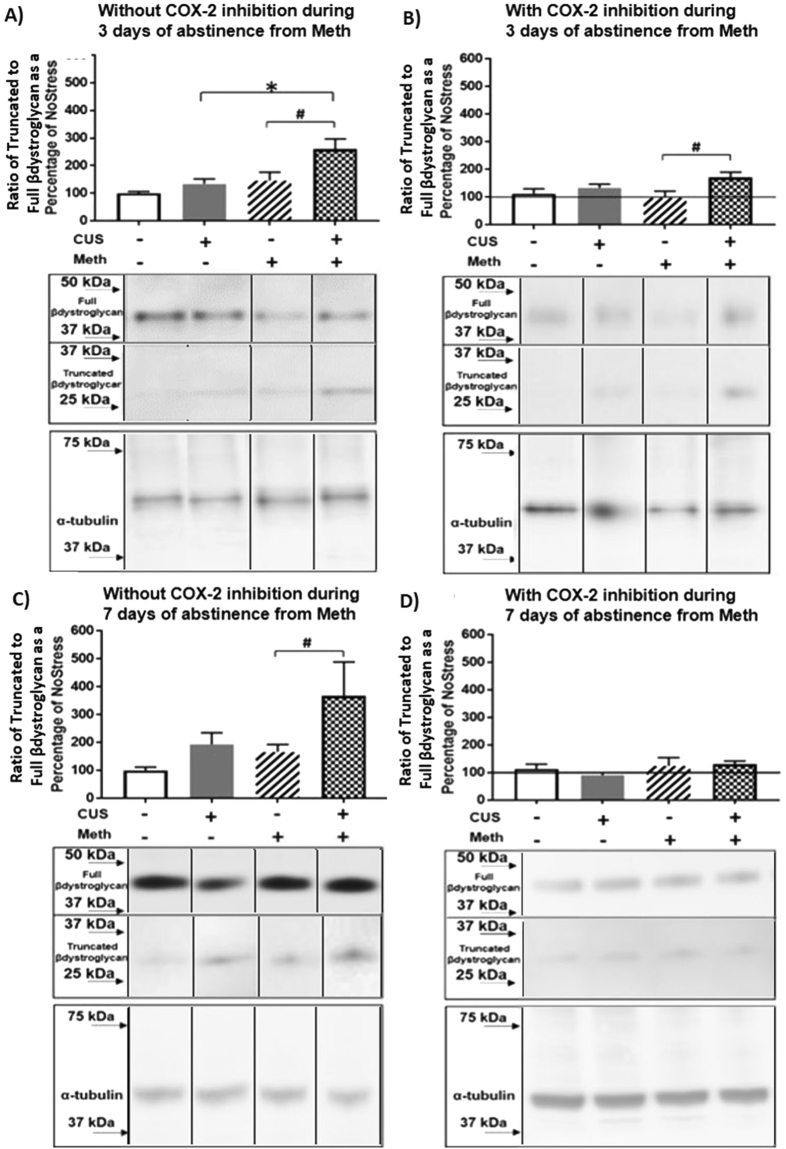
Truncation of β-dystroglycan in cortical microvasculature during abstinence from Meth. **(A**) 3 days of abstinence from Meth. CUS + Meth compared to CUS (*p < 0.01) and Meth (^#^p < 0.01) n = 7–8/group. Middle panel shows representative blot of full and truncated forms of β-dystroglycan after 3 days of abstinence from Meth and the bottom panel shows the loading control α-tubulin. (**B)** COX-2 inhibitor nimesulide treatment during 3 days of abstinence CUS + Meth vs. Meth (^#^p < 0.01) n = 6–8/group. Line indicates controls that did not receive nimesulide treatments. Middle panel shows representative blot of full and truncated forms of β-dystroglycan in nimesulide treated groups after 3 days of abstinence from Meth and the bottom panel shows the loading control α-tubulin. (**C**) 7 days of abstinence from Meth and β-dystroglycan in CUS + Meth vs Meth (^#^p < 0.01) n = 6/group. Middle panel shows representative blot of full and truncated forms of β-dystroglycan after 7 days of abstinence from Meth and the bottom panel shows the loading control α-tubulin. (**D**) COX-2 inhibitor nimesulide treatment during 7 days of abstinence. Line indicates controls that did not receive nimesulide treatments. Middle panel shows representative blot of full and truncated forms of β-dystroglycan after nimesulide during 7 days of abstinence from Meth and the bottom panel shows the loading control α-tubulin. β-dystroglycan and α-tubulin band images presented have been vertically sliced in order to juxtapose lanes that were non-adjacent in the blot. Full-length blots are presented in Supplementary Figures S4A, S4B, S4C and S4D.

Truncation of β-dystroglycan was examined at 3 days after Meth and was increased by CUS (F_(1,26)_ = 7.61, p < 0.05) and Meth (F_(1,26)_ = 10.10, p < 0.01). Specifically, β-dystroglycan was increased in the CUS + Meth group compared to CUS + NoMeth (q = 4.65, p < 0.01 CUS + NoMeth = 134.76 ± 27.98, CUS + Meth = 259.81 ± 26.00) and NoStress + Meth groups (q = 4.38, p < 0.01, NoStress + Meth = 146.01 ± 26.00) (Fig. 4A). Nimesulide treatment during Meth abstinence reduced the truncation of β-dystroglycan produced by CUS + Meth (F_(1,20)_ = 6.41, p < 0.05). Post-hoc comparison showed that truncation of β-dystroglycan was greater in the CUS + Meth vs NoStress + Meth (q = 3.95, p < 0.01 CUS + Meth = 171.28 ± 18.36, NoStress + Meth = 96.93 ± 24.48) (Fig. 4B). Additionally, 7 days after abstinence from Meth, the truncation of β-dystroglycan remained increased in CUS groups (F_(1,24)_ = 4.95, p < 0.05). In particular, β-dystroglycan in CUS + Meth was elevated compared to NoCUS + Meth treatment (q = 3.15, p < 0.05 CUS + NoMeth = 166.36 ± 61.36, CUS + Meth = 366.66 ± 65.59) (Fig. 4C). Nimesulide treatment administered during 7 days of abstinence from Meth significantly blocked the increases in β-dystroglycan (Fig. 4D).

## Discussion

This study demonstrates that chronic stress followed by voluntary Meth intake and abstinence alters the cerebral microvasculature. Results show that although CUS did not affect the self-administration of Meth, prior exposure to CUS increased COX-2 and breakdown of β-dystroglycan in cortical capillaries that persisted for 7 days after abstinence from Meth self-administration. Additionally, the serial exposure to CUS and Meth decreased the tight-junction protein occludin at 7 days after Meth abstinence. Furthermore, inhibition of COX-2 during only the period of abstinence from Meth blocked the breakdown of β-dystroglycan as well as decreases in occludin.

Self-administration studies have usually examined the effects of acute or repeated exposure to the same stressors on drug intake^20^; however these stress paradigms do not reflect the often variable nature of stress^29,30^. The present study employed a variable stress paradigm that approximates how humans are chronically exposed to stress. Furthermore, the current study was designed to allow for unrestricted access to Meth while controlling for total Meth intake by limiting the dose to 40 mg/kg. This design avoided the variations in total dosing inherent to typical self-administration studies where the animal is given unrestricted access to the drug for a fixed period of time, thereby producing greater variability in total Meth intake between animals. In contrast, the current design permits more clear interpretations to be made related to a given dose and changes in the BBB. Furthermore, limiting the total drug intake to 40 mg/kg permits comparison to published studies that typically use this dose in non-contingent, investigator-administered Meth paradigm to evaluate neurotoxicity and cerebrovascular injury^13,19,24^.

Stress is known to increase the self-administration of psychostimulants such as cocaine, amphetamine and nicotine^31–33^. In contrast, the current results indicate that CUS did not affect the voluntary administration of Meth (Fig. 1A). This could be due to the different type of stressor paradigm used in this study. Whereas a *mild* chronic unpredictable stress paradigm was used here, other studies have used repeated exposure to severe stressors such as restraint, tail-pinch or exposure to large aggressive counterparts^31,33^. Therefore, the less severe stress exposure employed in the current study could explain why stress did not alter Meth self-administration. Additionally, the absence of stress induced potentiation of Meth self-administration could be due to the unrestricted access to Meth that was allowed in this study compared to other studies where the duration of access to the drug is limited each day^34,35^. The lack of drug availability during the time the animal is not in the operant chamber could trigger withdrawal-associated processes that promote craving and increase drug administration in the stressed group^36^. It is also possible that concomitant rather than the serial exposure to mild CUS prior to Meth may have a greater impact on the self-administration of Meth. Interestingly, stress did not affect the ability of the rats to learn the procedure (Fig. 1B) even though CUS causes cognitive deficits^37^. Regardless, the finding that CUS did not alter Meth intake suggests that the effect of CUS is independent of the amount of Meth self-administered yet synergizes with Meth to alter BBB proteins.

Meth can compromise the blood-brain barrier (BBB) through neuroinflammation^13,19,38,39^ but these studies did not examine the effects of self-administration of the drug, the temporal pattern of changes produced by abstinence from Meth, and/or its relationship to CUS. The current results show that COX-2 protein in the cortical capillaries increased throughout the 7 day abstinence period, indicative of a sustained and persistent increase in COX-2 (Fig. 2A,B). The increases in COX-2 could be a compensatory response to decreased enzymatic function, however this is unlikely to be the case as increased COX-2 protein and COX-2 activity has been reported in BBB disruption due to ischemia and traumatic brain injury^40–42^. Thus, these findings indicate that prior exposure to chronic stress can sensitize a subsequent COX-2 dependent response^43,44^ and promote cerebrovascular disruption after Meth.

Few if any studies have attempted to relate BBB changes to markers associated with inflammation after varied periods of abstinence from voluntary exposure to Meth. The persistent increases in COX-2 protein that ensue after the sequential exposure to CUS and Meth resulted in a significant but delayed decrease in the BBB structural protein, occludin within cortical capillaries (Fig. 3A,C). The significant decrease at 7 days is in agreement with Northrop and Yamamoto^13^, wherein similar decreases in occludin were observed after serial exposure to stress and investigator administered Meth. The current study however, did not observe decreases in occludin after 3 days of abstinence from Meth. The more delayed response to occludin in the current study is likely due to the dispersion of Meth doses over a more extended period of time compared to the binge-like administration of bolus doses over a 6 h period^13^. Regardless, the decrease in occludin after 7 days but not 3 days of abstinence from Meth after exposure to CUS is consistent with sustained inflammatory changes during abstinence exhibited by Meth and cocaine abusers^11,45^. The current finding that COX-2 inhibition with nimesulide during abstinence from Meth blocked the decreases in capillary occludin (Fig. 3D) indicates that a COX-2-dependent mechanism during forced abstinence disrupts the cerebrovasculature.

The sequential exposure to CUS and Meth also disrupted β-dystroglycan, a transmembrane protein that cross-links vascular endothelial cells and astrocytic end-feet to the basement membrane^46^. The truncation of β-dystroglycan followed a similar time course to that of COX-2. Although the truncation of β-dystroglycan was blocked by nimesulide when administered over the 7 days, it was not blocked when administered over 3 days of abstinence, suggesting that a COX-2 independent mechanism may mediate the truncation during the first 3 days of abstinence from Meth. Regardless, breakdown of β-dystroglycan can free the endothelial cells from their anchor to the basement membrane and contribute to the destabilization between vascular endothelial cells and the eventual decrease in occludin protein mediated by COX-2 (Figs 4A and 3C).

COX-2 in vascular endothelial cells can activate multiple inflammatory pathways that lead to cerebrovascular toxicity. COX-2 mediates prostaglandin signaling that leads to increases in matrix metalloproteinase activity in the basement membrane as well as increases in pro-inflammatory cytokines and reactive oxygen species that can truncate β-dystroglycan, cleave occludin, and ultimately destabilize the BBB^47–49^. Furthermore, prostaglandins can increase phosphorylation of occludin via Rho kinase activity which leads to permeabilization of the BBB^50^. Therefore, the observed changes in COX-2 are suggestive of inflammation within the cortical capillaries mediated by multiple inflammatory pathways activated by CUS, contingent and voluntarily administered Meth, and abstinence from Meth, all of which converge to synergize and enhance COX-2 and destabilize components of the BBB.

Overall, this study demonstrates that serial exposure to relatively mild-to-moderate environmental factors that by themselves do not alter cerebrovascular structure, can synergize to cause persistent inflammation that eventually disrupts the BBB. The observed changes to the BBB however, do not occur during CUS or during Meth because there are no effects on the tight junction protein occludin after either CUS or during the first few days of abstinence from the self-administration of Meth. This delayed effect obviates the need for prophylactic treatment before or during stress and/or Meth exposure and offers a window of opportunity to pharmacologically intervene and prevent alterations to the BBB with a COX-2 inhibitor after CUS and Meth. In conclusion, this study provides promising evidence for the use of a COX-2 antagonist administration during forced abstinence from drug self-administration for the treatment of co-morbid effects of stress and Meth induced changes to the cerebrovasculature.

## Methods

### Animals

Experiments were conducted in compliance with the Guide for the Care and Use of Laboratory from the National Institutes of Health and approved by Indiana University Institutional Animal Care and Use Committee. Male Sprague-Dawley rats, 225–250 g were purchased from Envigo, and housed 2 per cage in a temperature and humidity controlled room under a 12 h light/dark cycle (6:00 am–6:00 pm). Food and water were available ad lib during a 1 week acclimation period prior to experimentation.

### CUS paradigm

Rats underwent CUS for 10 days or remained in the home cage (NoStress). CUS was used to prevent habituation to stress exposure and to model unexpected stressful circumstances that occur in humans^29,51^. The CUS paradigm is as follows: day 1: 60 min 4 °C cold exposure (10:00 h) and 3 min swim (14.30 h); day 2: 60 min social (11:30 h) and 60 min restraint (16:00 h); day 3: 3 h lights off (11:00 h) and lights on overnight (18:00 h); day 4: 30 min elliptical shaker (9:30 h) and 90 min wet bed (13:00 h); day 5: 60 min restraint (10:30 h) and 3 min swim (14:30 h); day 6: rats underwent jugular vein catheter implantation surgery; day 7: 30 min cold room (11:30 h) and 60 min wet bed (16:00 h); day 8: 30 min social stress (9:00 h) and 60 min shaker (13:00 h); day 9: 3 h lights off (10:30 h) and lights on overnight (18:00 h); day 10: 30 min 4 °C cold exposure (11:00 h) and 60 min shaker (13:30 h). For the swim stress, rats were placed in groups of 6–8 in a 15 × 10 × 24′ bucket containing 18′ of water maintained at RT. The restraint stress involved confining rats in a commercially available flat-bottomed plexiglass rodent restrainers (Harvard Instruments). During the shaker stress, the rats were placed in orbital shakers and rotated at 30 rpm. The wet bedding stress involved placing the rats in clean cages with bedding that was saturated with RT water. For the social stress, cage-mates were separated and exposed to a new male rat in a similar weight range for the duration of the stress.

### Jugular-vein Catheter Implantation Surgery

Rats were anesthetized with ketamine (75 mg/kg, Henry Schein, Cat. 56344) and xylazine (5 mg/kg Santa Cruz, Cat. sc362950Rx). Once unconscious, fur was removed from the chest and back by applying depilatory cream around the clavicle and the scapulae. For analgesia, rimadyl (0.1 cc of 5 mg/ml, sc) and buprenorphine (0.2cc of 0.05 mg/ml, sc., Henry Schein, Cat. 59122) was given to the rats. The exposed skin was wiped with 70% isopropanol and the rat was placed on a sterile drape (Fisher, Cat. 9004686) and the chest and back was scrubbed with betadine and 70% isopropanol. An incision was made along the clavicle and the underlying fascia was retracted to expose the jugular vein. A small incision was made on the vein and a polyurethane catheter (Access Technologies, Cat. AT-RJVC-0612A) filled with flushing solution (200 mg cefazolin (Fisher, Cat. AC455210010), 0.2 ml heparin (Henry Schein, Cat. 55737), 19.8 ml filtered saline) was inserted into the vein and secured in place by tying the catheter to the vein with sterile silk suture (Henry Schein, Cat. 1005597).The other end of the catheter was tunneled under the skin to exteriorize between the scapulae and connected to a port (Plastics One, Cat. 313-000BM-15-5UP/SPC) that was capped with a heat-sealed tygon tubing. The incisions were sutured closed and 2% lidocaine and triple antibiotic cream was applied to the wound. Catheter patency was tested and maintained by daily infusions of 0.1 cc flushing solution via the port until the rats were connected to the Meth self-administration chambers. Jugular vein catheters were also implanted in control rats but the rats were not placed in the self-administration chambers.

### Meth Self-Administration Procedure

One day after last CUS, rats were placed in sound-attenuating self-administration chambers (MedAssociates, Cat. ENV0008CTT) with a 6 am–6 pm light-on cycle and allowed unrestricted access to food, water and Meth until they reached 400 Meth rewards. The rats were allowed 7 days to reach the 400 rewards criterion. The self-administration chambers were equipped with an active and inactive lever with cue-lights above the lever. The port mounted on the back of the rat was connected to polyethylene (PE) tubing covered by a spring tether attached to a swivel on a counter balance arm. The other end of the swivel was connected via PE tubing to a syringe filled with Meth. The syringe was placed in an infusion pump that delivered 100 μl of 0.1 mg/kg Meth (FR1 schedule) over the course of 5 s when the active lever was pressed. During this period the cue-light above the active lever turned on to indicate a 5 s “time-out”, where additional active lever-presses did not result in a reward. Inactive lever presses were recorded but had no programmed consequences. Upon reaching the 400 rewards criterion, brains were extirpated and capillaries were isolated. The CUS controls remained in the home cage and were killed at time points matching the Meth self-administering groups.

### Drugs Used

(+) Methamphetamine (Meth) (Sigma, Cat. M-8750) was dissolved in 0.9% sterile saline containing 0.001% heparin (70 U/ml). The dose of Meth infusion was based on previous studies of self-administration and investigator-administered Meth^6,13,52^.

Nimesulide (7.5 mg/kg, ip, Cayman chemical, Cat. 70640) was injected twice daily as a suspension in 0.9% saline, once in the morning and once in the evening starting on the first day of abstinence when the rats were removed from the self-administration chamber. The dose and time-course of treatment was based on elimination half-life, toxicity and previous studies^41,53–57^.

### Cortical Capillary Isolation

Cortical capillary isolation was carried out based on established protocol by Yousif *et al*.^58^ and Northrop and Yamamoto^13^. The overlying cortex was removed from the rat brain and placed in a petri dish containing cold Hanks’ balanced salt solution (HBSS). The meninges and large superficial blood vessels were removed. The cortex was then placed in an eppendorf tube containing 500 μl HBSS and was minced and suspended by pipetting up and down with a 1 ml pipettor. The homogenate was centrifuged at 1000 RCF for 10 min after which the supernatant was removed and the pellet was resuspended in 500 μl of 17.5% dextran (Sigma Cat. 31390). This suspension was centrifuged at 4400 RCF for 15 min, the supernatant collected, and centrifuged again at 4400 RCF for 15 min. Both pellets were then suspended in HBSS, pooled and filtered through a nylon mesh. The filtrate was centrifuged at 1000 RCF for 10 min. The pellet containing the capillaries was suspended in 400 μl urea buffer (6 M urea, 10 mM Tris, 1 mM 1,4 dithiothreitol, 5 mM MgCl_2_, 5 mM EDTA-Na_4_, 150 mM NaCl and protease inhibitor cocktail, pH 8.0) and incubated at 4 °C over 2 nights. The sample was again centrifuged at 1000 RCF for 10 min and the supernatant was used for protein quantification (Bradford protein assay, BioRad, Cat. 500-0006).

### Western Blotting

Cortical capillary samples were diluted 1:4 with LDS sample buffer (Fisher, Cat. NP0007). Sample protein (20 µg for COX-2 and occludin; 60 µg for β-dystroglycan) was loaded onto 12% Bis-Tris gels (Invitrogen, Cat. NP0335BOX). Gels were run at 125 V for 2 h and transferred onto PVDF membranes at 29 V for 2 h. The membranes were blocked with 5% bovine serum albumin dissolved in 0.5% TBST (Tris-buffered saline (TBS) (10 mM Tris, 150 mM NaCl), containing 0.5% Tween-20for 1 h, incubated in M X occludin, 1:500, (Fisher, Cat. 331500), Rb X COX-2 1:500 (Fisher, Cat. 35-600-250UG), or M X β –dystroglycan 1:500, (Leica Microsystems, Cat. B-DG-CE)^13,16^ overnight at 4 °C. The next day, membranes were washed 3 × 5 min in 0.5% TBST, incubated at RT in horseradish peroxidase (HRP)-conjugated anti-mouse IgG 1:2500 (Santa Cruz, Cat. sc-2005) or Gt X rabbit IgG, 1:2500 (Millipore, Cat. AP307P) in blocking buffer for 1 h RT. Membranes were again washed 3 × 5 min in 0.5% TBST and incubated for 2 min in HyGLO enhanced chemiluminescence solution (Denville Scientific, Cat. E2500). LAS-4000 Image Analyzer (FujiFilm) was used to image and quantify optical density of proteins of interest. Membranes were then incubated in stripping buffer for 30 min (Fisher, Cat. 2502MI) followed by overnight incubation with the internal loading control α-tubulin (Sigma, Cat. T6074). A similar procedure as mentioned above was used to obtain the optical density of α-tubulin. Occludin, COX-2 and β-dystroglycan protein quantification were normalized to α-tubulin and results were calculated as a percentage of the control group. The breakdown of β-dystroglycan was calculated as the ratio of truncated to full form β-dystroglycan.

### Data Analysis

Data were analyzed using SigmaPlot 11.0. Two Way ANOVA with Stress and Meth being the main factors followed by post hoc Tukey’s multiple comparison tests were used for analysis of all data. Values are presented as mean ± SEM and significance was determined at p < 0.05.

### Data availability

The data generated and/or analyzed in this study are available from the corresponding author on reasonable request.

## Electronic supplementary material

Supplementary Information
