# Supplementary material for: Cerebrovascular Injury After Serial Exposure to Chronic Stress and Abstinence from Methamphetamine Self-Administration
Source: Sci Rep. 2018 Jul 12;8:10558. doi: 10.1038/s41598-018-28970-1 (PMC6043597; doi:10.1038/s41598-018-28970-1)

Cerebrovascular Injury After Serial Exposure to Chronic Stress and Abstinence from Methamphetamine Self-Administration

Reka Natarajan[1], Carmen M. Mitchell[1], Nicole Harless[2], and Bryan K. Yamamoto[1]*

[1] Department of Pharmacology and Toxicology, Indiana University School of Medicine
635 Barnhill Drive MS A401, Indianapolis IN 46202
[2] Department of Neurosciences, University of Toledo College of Medicine
3000 Arlington Avenue MS 1007, Toledo OH 43614

Corresponding Author: Bryan K. Yamamoto
Email: brkyama@iu.edu

Supplementary Figure 1

The bands presented in Figure 2 were obtained from the following blots. The boxes noted below identify the lanes used in the figures within the main paper.

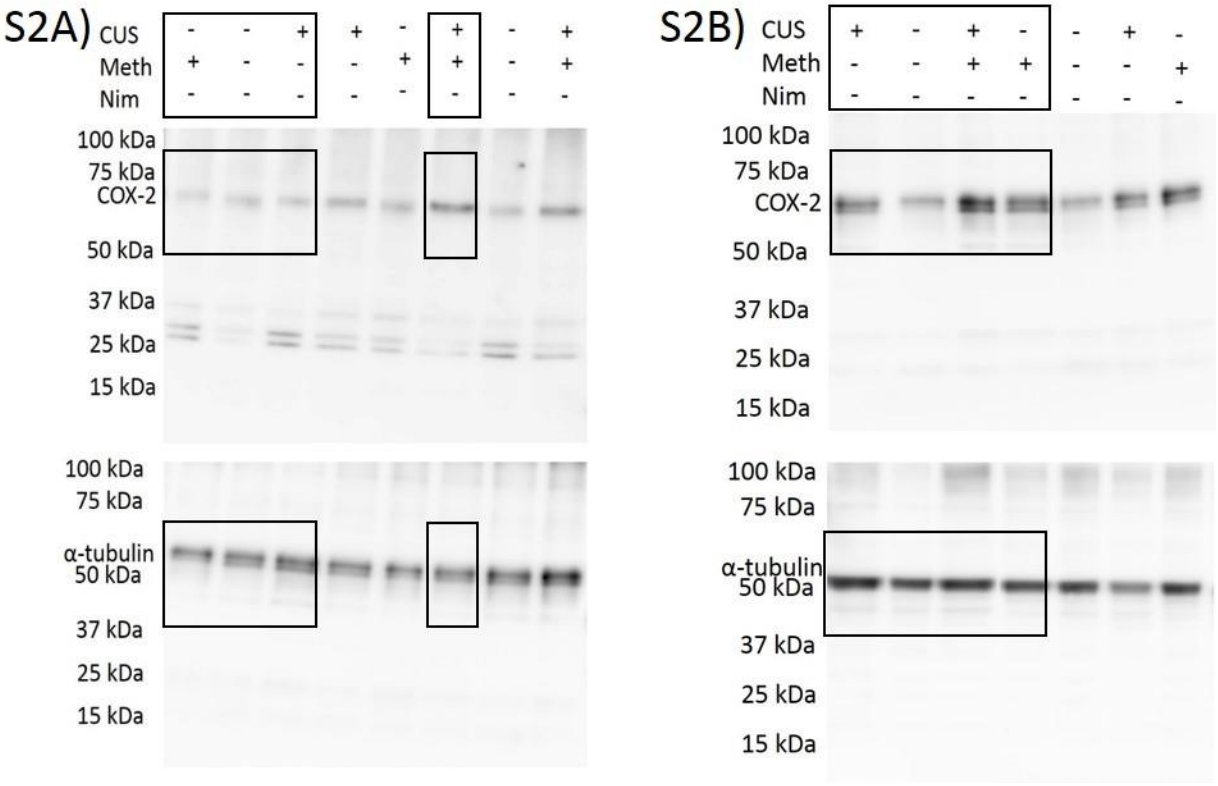

Supplementary Figure 2

The bands presented in Figure 3 were obtained from the following blots. The boxes noted below identify the lanes used in the figures within the main paper.

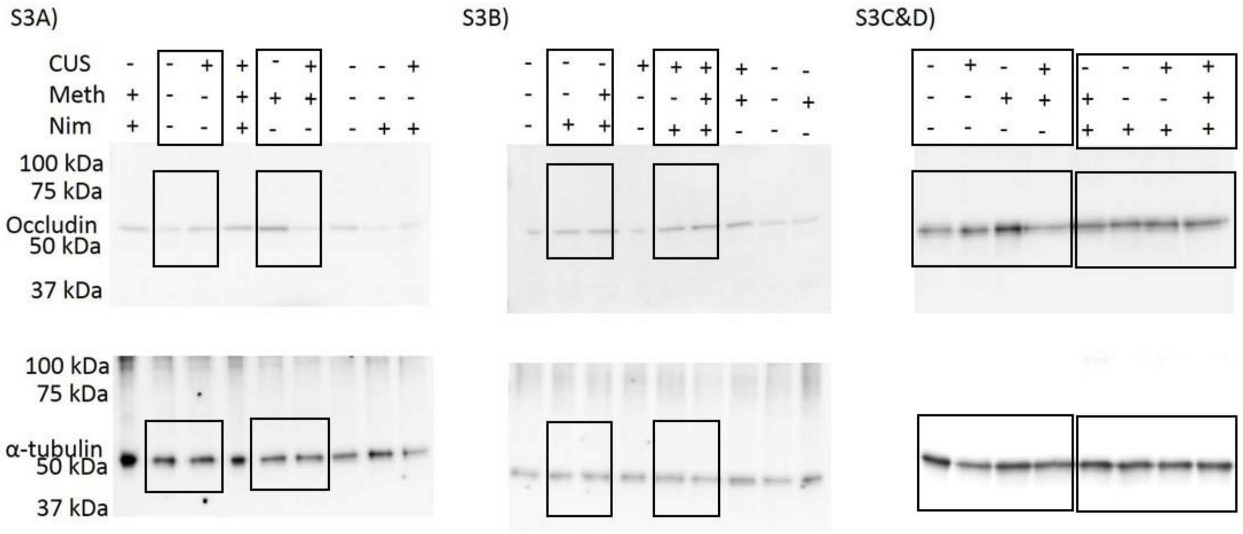

Supplementary Figure 3

The bands presented in Figure 4 were obtained from the following blots. The boxes identify the lanes used in the figures within the main paper. Short and long duration exposures corresponding to the full and truncated β-dystroglycan bands are presented in S4C and S4D and shown in Figure 4C and D of the main paper.

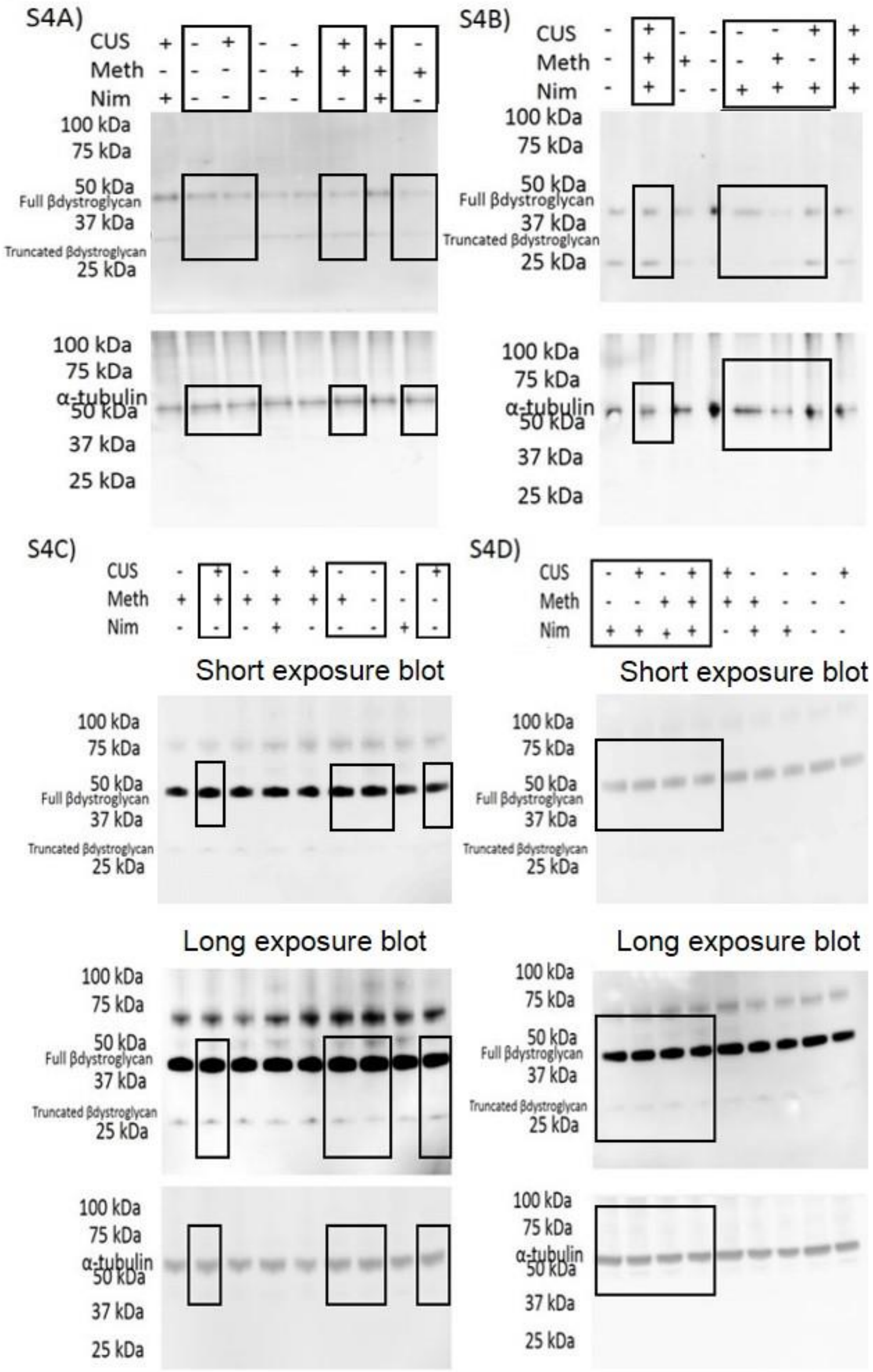

Supplement: Supplementary file 1 — Supplementary Information [file 41598_2018_28970_MOESM1_ESM.pdf]
